# Unidirectional molecular assembly alignment on graphene enabled by nanomechanical symmetry breaking

**DOI:** 10.1038/s41598-018-20760-z

**Published:** 2018-02-05

**Authors:** Liu Hong, Taishi Nishihara, Yuh Hijikata, Yuhei Miyauchi, Kenichiro Itami

**Affiliations:** 1 JST-ERATO, Itami Molecular Nanocarbon Project, Nagoya University, Chikusa Nagoya, 464-8602 Japan; 2 Graduate School of Science, Nagoya University, Chikusa Nagoya, 464-8602 Japan; 3 School of Chemical and Material Engineering, Jiangnan University, Wuxi, 214122 China; 4 Institute of Transformative Bio-Molecules (WPI-ITbM), Nagoya University, Chikusa Nagoya, 464-8602 Japan; 5 Institute of Advanced Energy, Kyoto University, Uji Kyoto, 611-0011 Japan

## Abstract

Precise fabrication of molecular assemblies on a solid surface has long been of central interest in surface science. Their perfectly oriented growth only along a desired in-plane direction, however, remains a challenge, because of the thermodynamical equivalence of multiple axis directions on a solid-surface lattice. Here we demonstrate the successful fabrication of an in-plane, unidirectional molecular assembly on graphene. Our methodology relies on nanomechanical symmetry breaking effects under atomic force microscopy tip scanning, which has never been used in molecular alignment. Individual one-dimensional (1D) molecular assemblies were aligned along a selected symmetry axis of the graphene lattice under finely-tuned scanning conditions after removing initially-adsorbed molecules. Experimental statistics and computational simulations suggest that the anisotropic tip scanning locally breaks the directional equivalence of the graphene surface, which enables nucleation of the unidirectional 1D assemblies. Our findings will open new opportunities in the molecular alignment control on various atomically flat surfaces.

## Introduction

Precise formation of on-surface nanostructures at the molecular level has long been of central interest in surface science^1–5^. Since the first direct observations of well-organized molecular assemblies on surface using scanning probe techniques^6^, orientation control of such assemblies has been intensively studied because of their importance in chemistry^1,5,7^, electronics^2,3,8^, biomimetics^4^, and optics^9,10^. There have also been a number of reports on polymerization of well-oriented mesoporous silica thin films using on-surface assembly of surfactant molecules as templates^11^. In addition, modulation of the electronic or optical properties of the two-dimensional (2D) material substrates, including graphene and related 2D semiconductors^12,13^, via interactions with molecular assemblies has recently emerged as a new interdisciplinary research field over surface chemistry and nanomaterials science. Thus, the development of the precise on-surface molecular assembly alignment method has become increasingly important.

The growth mode of molecular assemblies on solid surfaces is determined by delicate balances among various factors such as surface substrate properties^1,14,15^, molecular concentration^16^, solvent properties^17,18^, ionic properties^19^, and substrate friction^20^. In particular, the orientations of *in-plane* molecular assemblies are strongly affected by the symmetry of the substrate lattice because individual molecules tend to be first adsorbed with orientations along the well-defined axes on the surface^14,21–23^. Although precise orientation control is essential for various applications, *perfectly unidirectional* formation remains a challenge because of the surfaces normally have multiple axis directions with thermodynamical equivalence and one of them is just randomly selected for nucleation. Consequently, a typical in-plane molecular assembly on a surface has multiple domains with various orientations^22,24,25^. To lower the surface symmetry, researchers have proposed applying electric/magnetic fields^26^, using a macroscopic shear flow^27^, and controlling the surface boundary shape^28^. However, for the single-domain molecular assembly formation along a desired direction at any demanded position, further development of a simple and effective methodology of local surface symmetry breaking has still been strongly desired.

Here, we report the successful fabrication of an in-plane, unidirectional molecular assembly on graphene. We use nanomechanical symmetry breaking effects induced by scanning with the probe tip of an atomic force microscope (AFM); the inherently directional tip scanning is expected to strongly modify the surface thermodynamical conditions as well as to introduce effective anisotropy. This concept is distinctive from that previously proposed for mechanical nanomanipulation, where a quasistatic AFM tip operation was used to cut molecules or to move them to a desired position^29–31^. Assembly of typical surfactant molecules, sodium dodecyl sulfate (SDS), on graphene-water interface was studied as a prototypical case. Under the AFM tip scanning condition, we found that ribbon-like one-dimensional (1D) assemblies of the SDS molecules were generated. The orientation of the 1D molecular assemblies showed clear correlation with the symmetry axis of the graphene lattice and the AFM tip scan direction. The computational simulations proved that the AFM tip scanning led to the anisotropic adsorption stability of the molecules on graphene surface. On the basis of these understandings, we eventually achieved the unidirectional formation of the 1D molecular assemblies along a selected symmetry axis after removing the initially adsorbed molecules, by sequential fine tunings of the scan parameters. These findings offer new opportunities for the on-demand unidirectional molecular assembly alignment on atomically flat 2D surfaces, and shed light on the usefulness of the anisotropic nanomechanical effects in the AFM tip scanning, which has rather been considered as undesirable in conventional AFM observations.

## Results and Discussion

**Figure 1:**
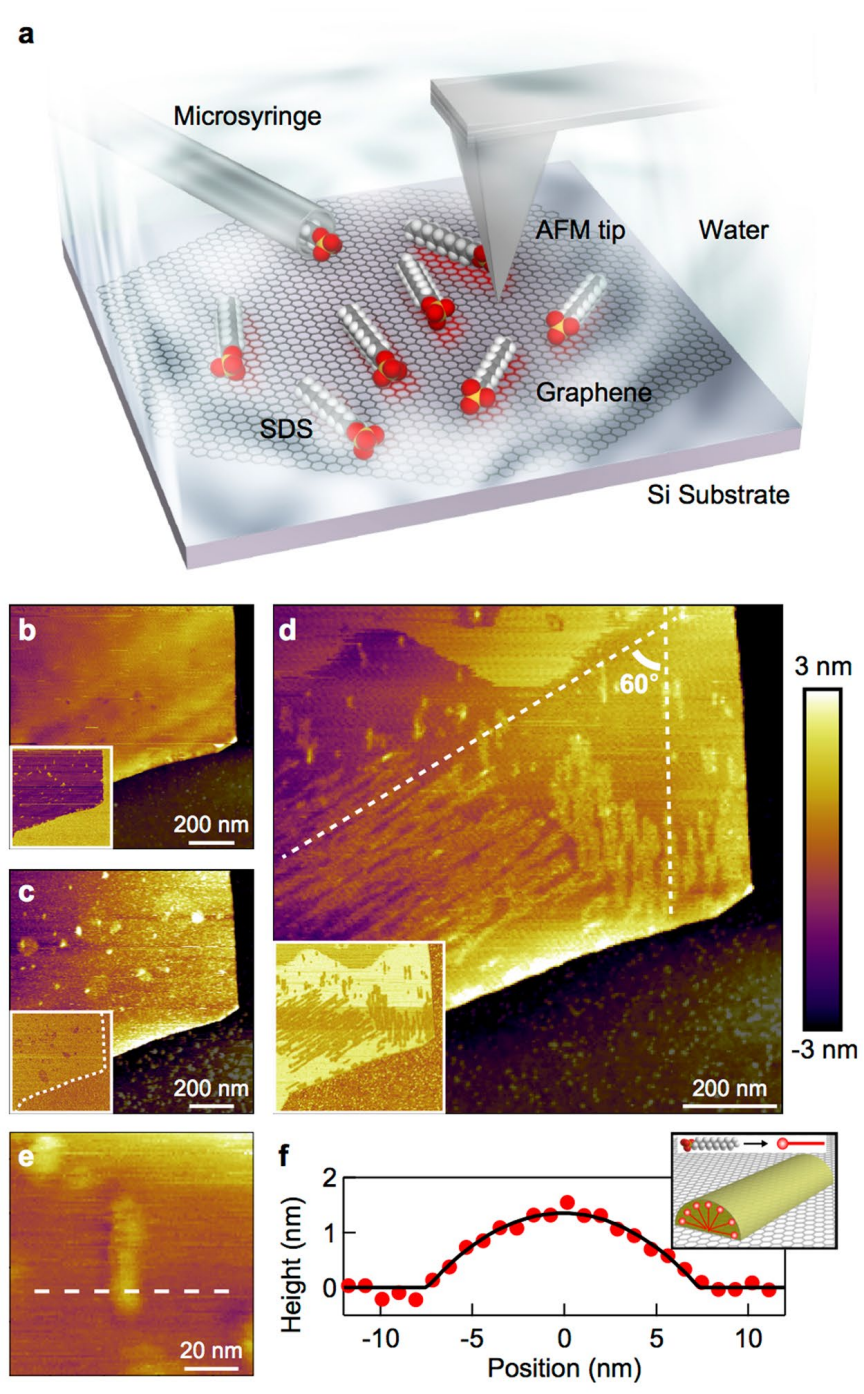
Effects of AFM tip scanning. (**a**) Schematic of the experimental setup. The SDS molecules and the graphene lattice are drawn enlarged for clarity. (**b,c,d)** AFM height images on multilayer graphene in water without the SDS molecules (**b**), 1 h after the injection of the SDS aqueous solution (**c**), and after 15 min of intense AFM scanning (**d**). For clarity, the silicon substrates are dark-colored. The AFM parameters were a tip–sample force (*f*_ts_) of 20 pN, a scan velocity (*v*_scan_) of 4.3 μm/s for the image acquisition, and an *f*_ts_ of 30 pN and a *v*_scan_ of 55.7 μm/s for the intense AFM scanning. The insets show the corresponding phase images. (**e)** Magnified image of a typical SDS ribbon on the graphene. (**f**) Height cross-section profile along the dashed line in (**e)**. The solid curve is the fitting result assuming a hemicylinder structure shown in the inset.

To demonstrate our concept, we first examined the effects of AFM tip scanning by comparing molecular adsorption morphologies at a liquid–solid interface with and without tip scanning. Figure 1a shows a schematic of the experimental setup. Multilayer graphene on a silicon substrate was placed in a water droplet, into which the SDS solution was slowly injected using a microsyringe pump. Figure 1b shows an AFM image of the graphene surface in water before the SDS injection. The AFM scanning was performed along the horizontal axis in the image. At this stage, no distinct structure was evident on the surface. We then injected the SDS solution. Figure 1c shows an AFM image recorded 1 h after the SDS solution injection. The height of the graphene increased by ~2.5 nm overall, and aggregates of various size appeared (see Supplementary Fig. S1). This result indicates spontaneous random adsorption of the SDS molecules onto the graphene surface after the SDS solution injection.

The subsequent intense scanning with a large force between the tip and sample (see Methods) drastically modified the SDS adsorption morphology; the initially adsorbed SDS molecules were removed, and new patterned structures emerged on the surface (see Supplementary Movie S1, Supplementary Note 1, and Supplementary Fig. S2). Finally, many ribbon-like molecular patterns (SDS ribbons) appeared (Fig. 1d). The cross-sectional height profile (Fig. 1f) of a typical ribbon (Fig. 1e) implies that the SDS molecules adopted the hemicylinder-like structure commonly observed for surfactant molecules adsorbed onto graphite^14,21,24^ (see Supplementary Note 2 and Supplementary Fig. S3). The hydrophobic end of the SDS molecules faces toward the graphene, whereas the hydrophilic end interacts with water (see inset of Fig. 1f). Notably, these ribbons can be grown individually, which qualitatively differs from the densely-packed assemblies previously observed under critical micelle conditions^14,24^. Under the conditions used in the present study, the SDS ribbons organized in multiple domains with different orientations, the relative orientation angle among which was approximately 60° (dotted lines in Fig. 1d). The angle of 60° was frequently observed in the AFM scanning experiments in this study (see Supplementary Note 3 and Supplementary Table S1), suggesting that the SDS molecules were adsorbed in an orientation reflecting the six-fold rotational symmetry of the underlying graphene lattice. Once SDS ribbons were stably grown, their orientations did not change with the subsequent change of the AFM scan direction and the scan rate (see Supplementary Note 4 and Supplementary Fig. S4).

**Figure 2:**
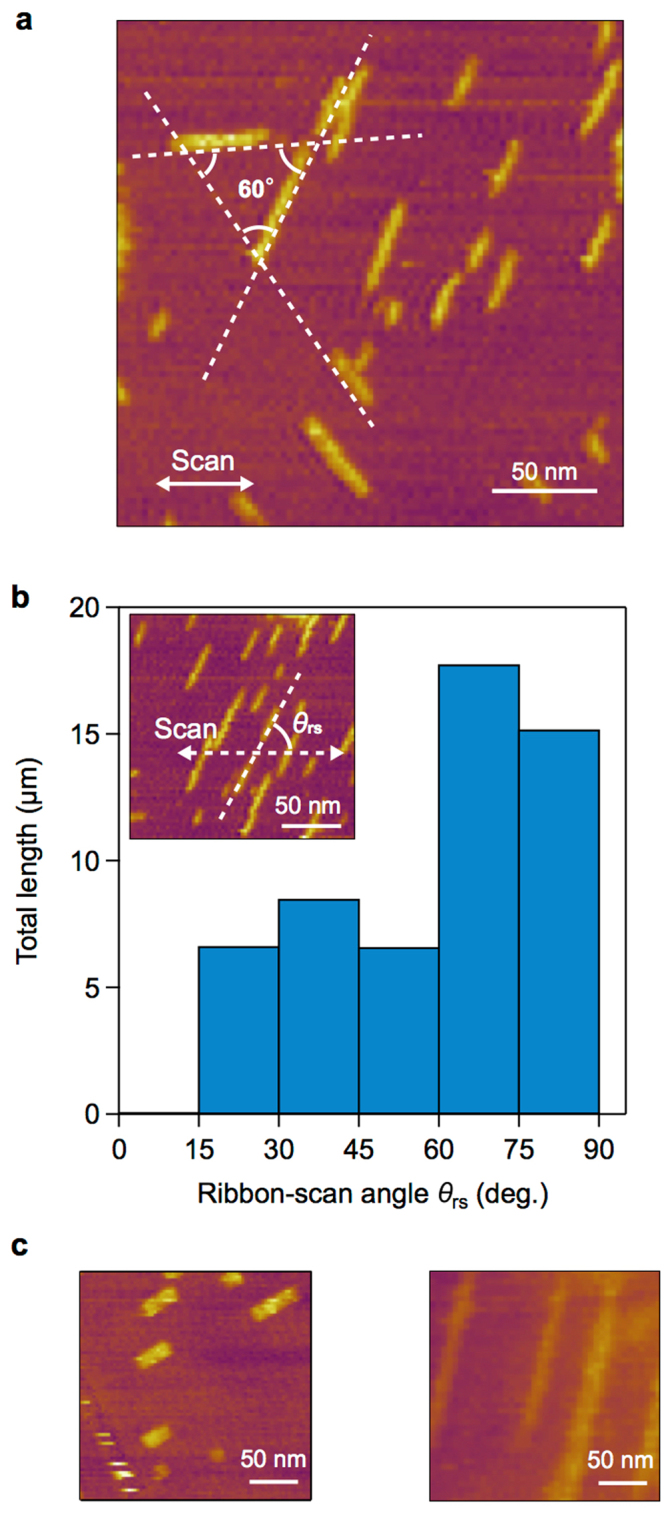
Statistical analysis of the ribbon orientation. (**a**) The typical relative orientations of the SDS ribbons. (**b**) The total length of the SDS ribbons as a function of ribbon-scan angle (*θ*_rs_). The inset of the AFM image shows the definition of the ribbon-scan angle *θ*_rs_. (**c**) Example of the SDS ribbons with *θ*_rs_ = 32° (left image) and 77° (right image).

We here focus on the orientations of the generated SDS ribbons in detail. Hereafter, the intense tip scanning operations were performed 15 min after the SDS injection in all of the experiments, which enabled us to examine the effects of the intense scanning in both the nucleation and growth of the ribbons more clearly (see Supplementary Movie S2 for the quasi-real-time observation of the nucleation and growth of the ribbons). Figure 2a shows a typical example of the formation of the SDS ribbons, where three preferable orientations of the SDS ribbons are observed and where each ribbon-to-ribbon angle is approximately 60°. In Fig. 2a, ribbons with their longitudinal axes parallel to the scan direction appear to be scarce. Because the six in-plane directions (or three axes) on the graphene surface should be thermodynamically equivalent as a consequence of the six-fold rotational symmetry, no preferential orientation of the ribbons is expected in the absence of the symmetry breaking effect by the AFM tip scanning. Thus, the results in Fig. 2a imply that the effect of the horizontal AFM tip scanning on the orientation of the generated SDS ribbons is non-negligible. To further examine this effect, we conducted statistical analyses of the correlation between the AFM scan direction and the observed ribbon orientation. We defined the relative angle between the ribbon longitudinal axis and the scan direction as the ribbon-scan angle *θ*_rs_ (0° ≤ *θ*_rs_ ≤ 90°), as shown in the inset of Fig. 2b. Figure 2b shows a histogram of the total length of the SDS ribbons observed in independent 291 scanning experiments. Even though the lattice directions of the graphene flakes used in each scanning experiment were undefined (random), the histogram clearly shows that the observed ribbon longitudinal axes and the AFM scanning direction are strongly correlated. Ribbons with *θ*_rs_ < 15° were rarely observed, and those with *θ*_rs_ > 60° exhibited the longest total length (see the typical ribbons in Fig. 2c).

**Figure 3:**
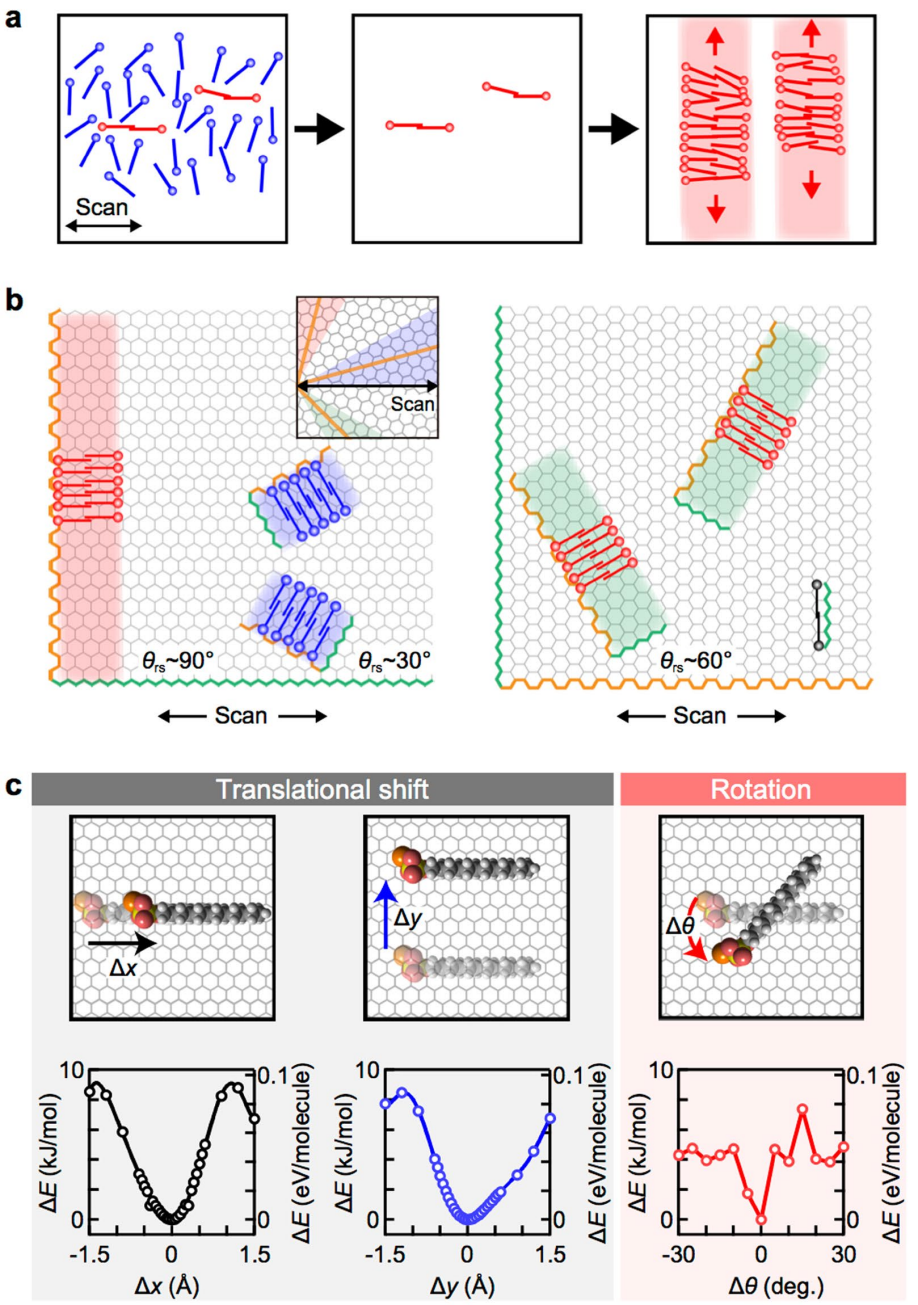
Formation mechanism of the oriented ribbons. (**a**) Schematic of the mechanism of selective removal of the SDS molecules followed by the ribbon growth process. (**b**) Preferential growing direction (indicated by red-shaded rectangles) of the SDS ribbons on the graphene lattice. The scan directions are parallel (left) and perpendicular (right) to the graphene zigzag (indicated by the green color) direction. Armchair directions are indicated by the orange color. The graphene lattice is drawn enlarged for clarity. The inset shows another graphene configuration. In any graphene configurations, the ribbons along the three graphene armchair axes (indicated by the orange lines) exhibit the ribbon-scan angles within the range of 0°–30° (blue), 30°–60° (green), and 60°–90° (red). The black- and blue-colored SDS molecules may be nuclei for ribbons, but they cannot grow into long ribbons (blue-shaded rectangles in the left panel and no black rectangle in the right panel) because of the efficient removal of the molecules with large relative angles to the scan direction. (**c**) Schematic of simulated molecular configurations with its longitudinal along zigzag direction (the upper panels). Calculated destabilization energies (Δ*E*) as functions of in-plane translational shifts (Δ*x* and Δ*y*) and rotation angle (Δ*θ*) of the SDS molecule (the lower panels).

Figure 3a illustrates a hypothetical process for SDS ribbon formation with preferential orientations; the underlying graphene lattice is neglected for simplicity. First, nuclei of the hemicylinder-like molecular assembly are randomly generated at the initial stage. Among these molecules, those adsorbed with relatively large angles to the scan direction are removed during the AFM tip scanning; only the molecules adsorbed with small angles to the tip scan direction survive. This process results in the growth of the ribbon with large *θ*_rs_ by side-by-side assembly of the SDS molecules^23^ (indicated by red-shaded areas in the right panel in Fig. 3a). Moreover, according to a previous study^23^ on the macroscopic ordering mechanism of the SDS assembly arrays on graphite, as the ribbon grows longer, the interaction between the ribbon and the graphene substrate becomes stronger, and the ribbon is expected to be further stabilized.

This hypothesis reasonably explains various aspects of the observed results. Figure 3b shows two simple cases where the scan direction is parallel (left) or perpendicular (right) to the graphene zigzag directions. Because our computational simulations (see Supplementary Note 5 and Supplementary Fig. S5) and previous studies^14,23^ show that an individual SDS molecule itself favors adsorption along the graphene zigzag directions, the growth direction of the ribbons should be perpendicular to the zigzag directions, that is, the graphene armchair directions (Fig. 3b). The molecules indicated by the red color have relatively small angles to the scan direction. If these molecular configurations enable the most efficient nucleation and the others (indicated by blue and black colors) are efficiently removed, the SDS ribbons preferentially grow nearly perpendicular to the scan directions. Although the lattice orientations of the graphene flakes in each experiment were random, in any graphene configuration, the angles of the three graphene armchair directions measured from the scan direction fall in the ranges of 0°–30°, 30°–60°, or 60°–90°, respectively (the inset of Fig. 3b). In addition, the angle between the ribbons on the same graphene surface should always be 60°. On the basis of the above consideration, the ribbon length is expected to be longer as the ribbon-scan angle increases and the ribbon-to-ribbon angle is expected to be 60°; the experimental results shown in Fig. 2a,b are consistent with these expectations.

To verify the aforementioned hypothesis, we examined the adsorption stability of SDS molecules on graphene using computational simulations. In general, the AFM tip scanning induces a finite frictional dragging force between the tip and the surface molecules because of microscopic shear flow or because of direct contact between the tip and the molecules^32^. This dragging force potentially induces translational or rotational motions of the adsorbed molecules, which could lead to their destabilization and desorption. We evaluated the destabilization energy of an SDS molecule on a graphene surface as functions of in-plane translational shifts and angular rotations using the self-consistent charge density-functional tight-binding method (see Supplementary Note 5 and Supplementary Fig. S5 for details). In the simulations, an SDS molecule was first positioned at the most stable site, with its orientation along the zigzag direction on a graphene lattice. We then calculated the destabilization energies as functions of the in-plane translational shift and rotation of the SDS molecule, as illustrated in Fig. 3c. The destabilization energy shows a quadratic dependence on both of the translational shifts, which means that the SDS molecules are stable against a small translational shift both along and perpendicular to the zigzag direction. By contrast, even a slight rotational shift causes considerable destabilization, which suggests that the SDS molecules are easily removed when forced to rotate under the AFM scanning conditions. The scan-induced rotational torque should be the smallest in the configuration where the molecular longitudinals are oriented in the scan direction because of its linear shape with a large aspect ratio. We thus conclude that the selective removal of the nuclei molecules of the ribbons is enabled through the rotational shift forced by the directional AFM tip scanning; this selective removal effectively breaks the symmetry on the graphene surface for the adsorbed SDS molecules.

**Figure 4:**
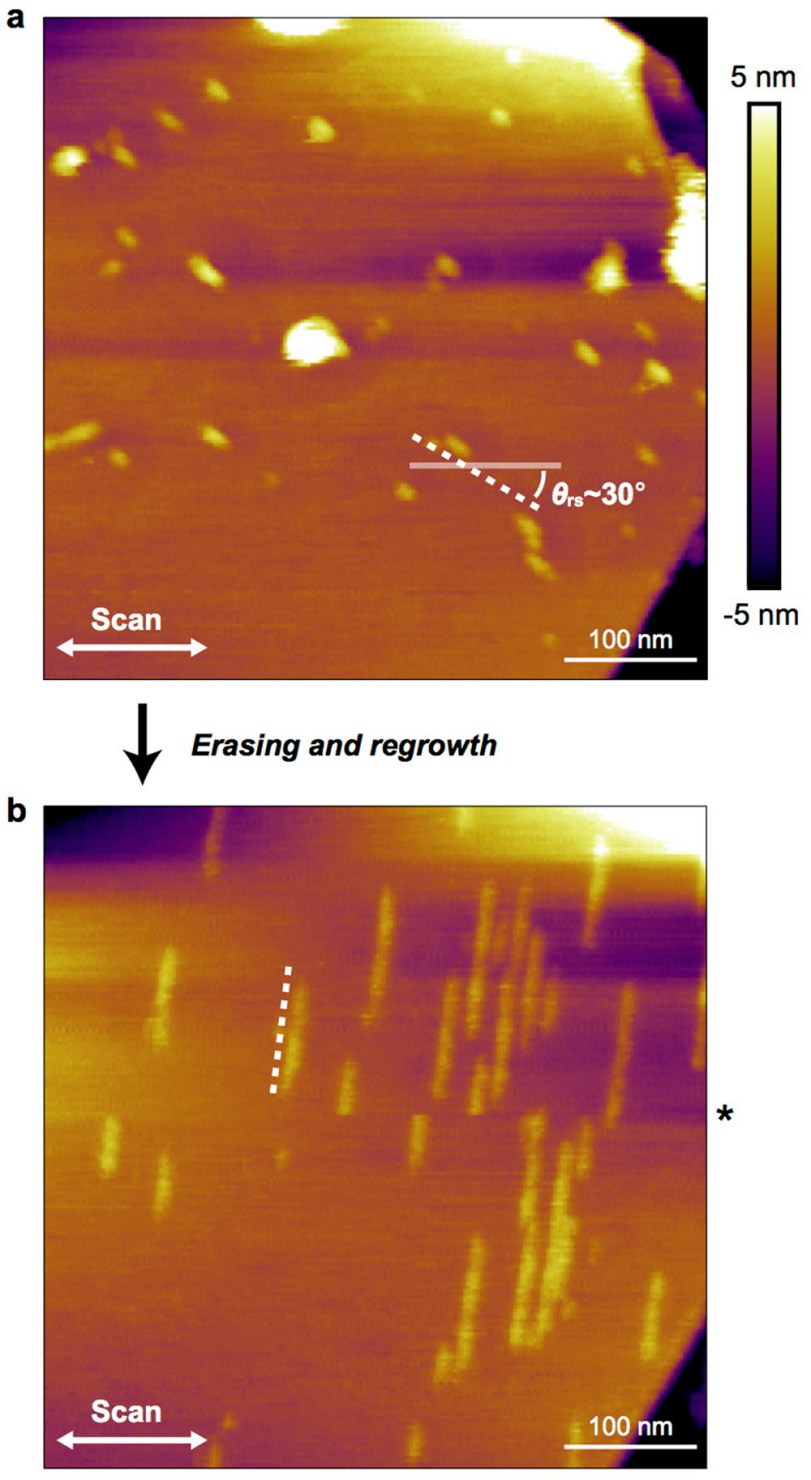
Unidirectional formation of the ribbons. (**a**) AFM image of the SDS ribbons formed under the scanning with an *f*_ts_ of 27 pN. (**b**) AFM image of the SDS morphology after the tip scanning with a strong *f*_ts_ of 42 pN. This image was recorded with an *f*_ts_ of 27 pN. One noise line (horizontal) was eliminated at the position indicated by an asterisk. The dotted lines indicate preferred orientations of the SDS ribbons.

On the basis of the aforementioned understandings, we demonstrate perfectly unidirectional formation of the SDS ribbons through sequential fine-tuning of the tip–sample force. Figure 4a shows an AFM image of a graphene surface in an SDS solution; relatively short SDS ribbons with *θ*_rs_ ≈ 30° are observed. This situation is similar to the case shown in the left panel of Fig. 3b, which indicates that the scan direction was nearly parallel to one of the zigzag directions. Under such conditions, we predict that the SDS ribbons with *θ*_rs_ ≈ 90° are more stable than those with *θ*_rs_ ≈ 30°, although, at this stage, no ribbons with *θ*_rs_ ≈ 90° are evident in Fig. 4a. We next performed AFM tip scanning while further increasing the tip–sample force. Consequently, as shown in Fig. 4b, all of the initially observed short SDS ribbons were removed (via erasing or nanoshaving^33^), and only the SDS ribbons with *θ*_rs_ ≈ 90° emerged on the same graphene surface (regrowth). This successful erasing–regrowth operation clearly demonstrates that one specific lattice direction can be selected for the ribbon growth among the multiple thermodynamically equivalent directions on the graphene surface. This result also clearly demonstrates that the presented mechanism concerning the stability of the adsorbed molecules is valid because the ribbons with *θ*_rs_ ≈ 90° were more stable than those with *θ*_rs_ ≈ 30°. The presented method for the precise unidirectional formation of individual molecular assembly nanostructures may enable the on-demand fabrication of more complicated and functionalized molecular nanostructures.

## Conclusions

In summary, we demonstrated the formation of in-plane-oriented molecular assembly nanostructures under fine-tuned AFM tip scanning conditions. The SDS molecules were assembled into ribbon-like nanostructures at the water–graphene interface, with well-defined in-plane orientations determined by both the underlying graphene lattice and the AFM tip scan direction. Moreover, the unidirectional formation of the ribbons was achieved via sequential fine-tuning of the tip–sample force. The angle-selective destabilization through angular rotation of the adsorbed molecules forced by the AFM tip scanning was proposed as the possible origin of the directional molecular assembly formation, as supported by experimental statistics and computational simulations. These results will pave the way for the on-demand formation of molecular nanostructures at a desired position and direction on various atomically flat solid surfaces.

## Methods

The SDS molecules were dissolved in water (0.6 wt%), and multilayer graphene flakes were transferred onto silicon substrates. We used this concentration because only the SDS aggregations were observed at higher concentration (2.0 wt%). An AFM probe tip and multilayer graphene on a silicon substrate were placed in a water droplet, into which the SDS solution could be slowly injected at a rate of 75 μL/h using a microsyringe pump. Consequently, the concentration of SDS molecules reached approximately 1.36 mM. All of the measurements were performed at room temperature.

The AFM measurements were carried out in “tapping in fluid” mode using a Dimension FastScan AFM system (Bruker, Santa Babara, CA, USA). In the examination of the effect of AFM tip scanning, the AFM images (Fig. 1b,c, and d) were obtained with a tip–sample force (*f*_ts_) of 20 pN (see Supplementary Note 6 for the detailed estimation method) and a scan velocity (*v*_scan_) of 4.3 μm/s. The intense tip scanning for generating ribbons was performed in 15 min with an *f*_ts_ of 30 pN and a *v*_scan_ of 55.7 μm/s. In independent 291 scanning experiments, the scanning time was set as about 20 min. In the demonstration of unidirectional ribbon formation, the AFM images (Fig. 4a,b) were collected with an *f*_ts_ of 27 pN and a *v*_scan_ of 1.3 μm/s. The unidirectional ribbon formation was achieved with an *f*_ts_ of 42 pN and a *v*_scan_ of 32.4 μm/s.

## Electronic supplementary material

Supplementary Information

SupplementaryMovie_S1.mov

SupplementaryMovie_S2.mov
