# Supplementary material for: Unidirectional molecular assembly alignment on graphene enabled by nanomechanical symmetry breaking
Source: Sci Rep. 2018 Feb 5;8:2333. doi: 10.1038/s41598-018-20760-z (PMC5799215; doi:10.1038/s41598-018-20760-z)
Supplement: Supplementary file 1 — Supplementary Information [file 41598_2018_20760_MOESM1_ESM.pdf]

## SUPPLEMENTARY INFORMATION

### Unidirectional molecular assembly alignment on graphene enabled by nanomechanical symmetry breaking

Liu Hong, Taishi Nishihara, Yuh Hijikata, Yuhei Miyauchi, and Kenichiro Itami

## Supplementary Figures

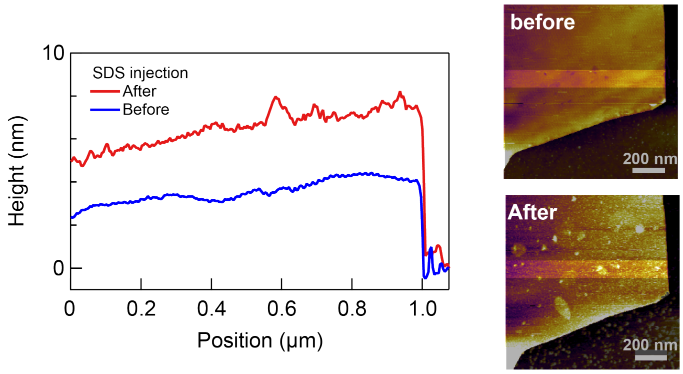


**Supplementary Figure S1.** Average height profiles on the graphene picked from the highlighted area in the corresponding AFM images (right panels). The data in the right panels are the same as those in Figs. 1b and 1c in the main text. Blue and red curves correspond to the height profiles before and after the SDS injection, respectively.

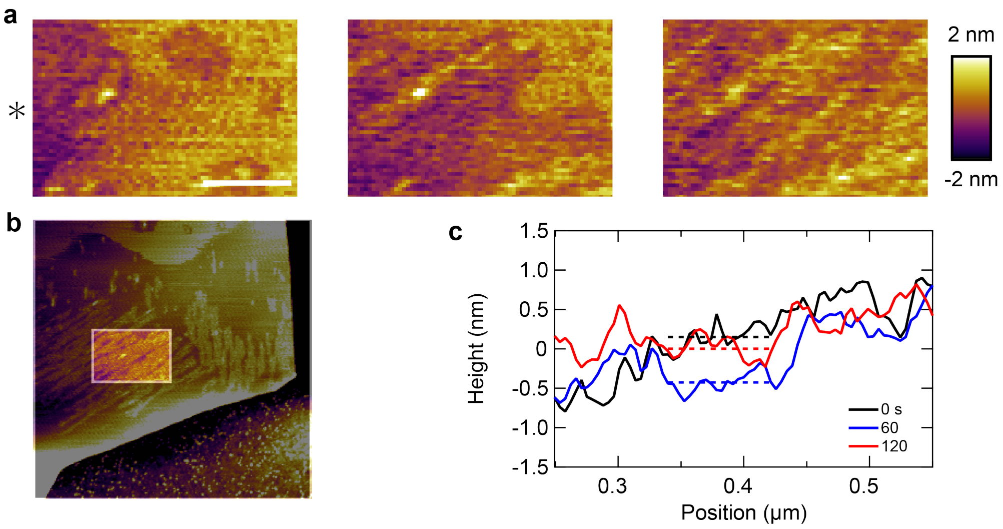


**Supplementary Figure S2. a,** The AFM images recorded at 0 (left), 60 (central), and 120 s (right) during the intense tip scanning. The scale bar is 100 nm. **b,** The highlighted rectangle indicates the area shown in **a**. **c,** The cross-section profile along the horizontal axis at the position indicated by the asterisk in **a**.

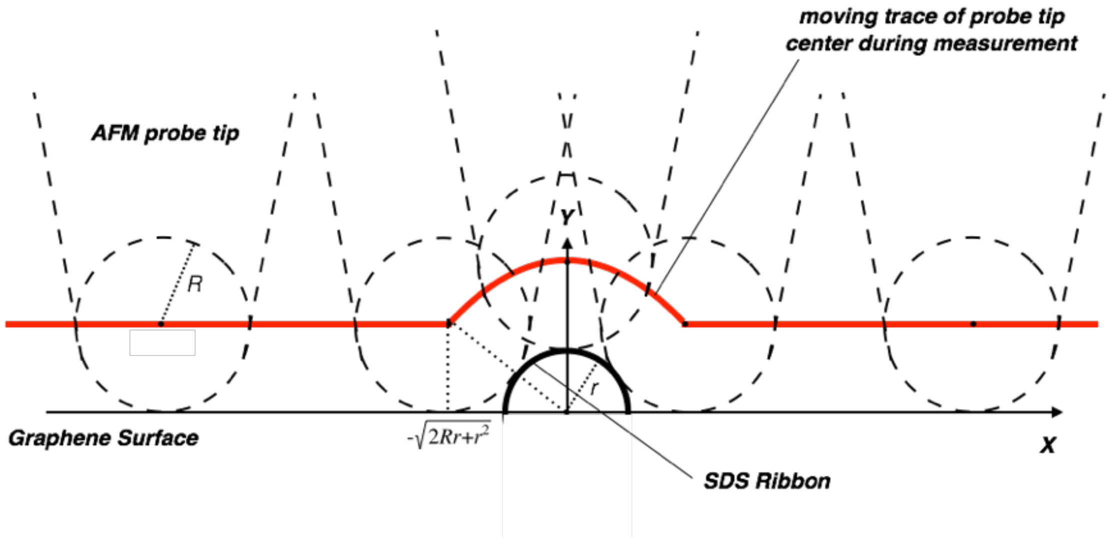


**Supplementary Figure S3.** Schematic of the AFM probe tip (dashed line)–hemicylinder (solid line) model used for fitting of the ribbon cross section to the hemicylinder structure. The moving trace of the probe tip center is indicated by the red curve.

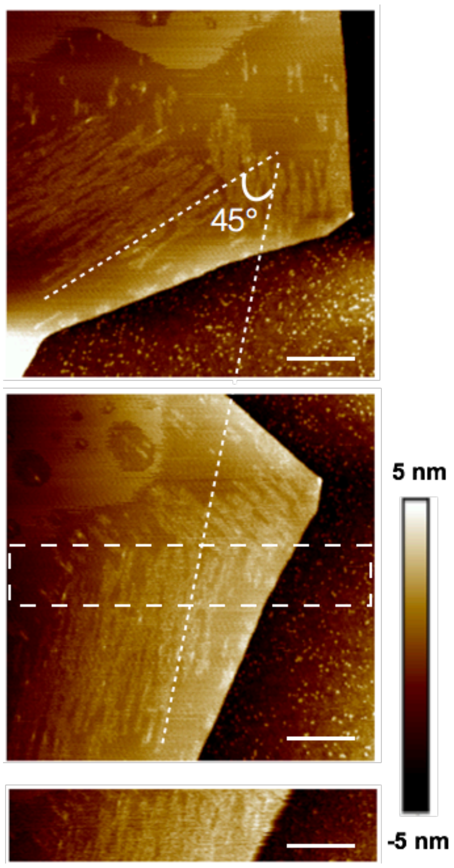


**Supplementary Figure S4**. Upper: AFM image of SDS/graphene sample with a horizontal scan direction. Scan rate: 4.3 μm/s. Middle: AFM image of SDS/graphene sample with a scan direction 45-degree rotated with respect to horizontal axis. Scan rate and other parameters were kept identical with the ones used for upper image. Lower: AFM image of SDS graphene sample recorded at a smaller area (dotted box in middle image) with a scan rate of 55.7 μm/s. Scan bar size: 200 nm.

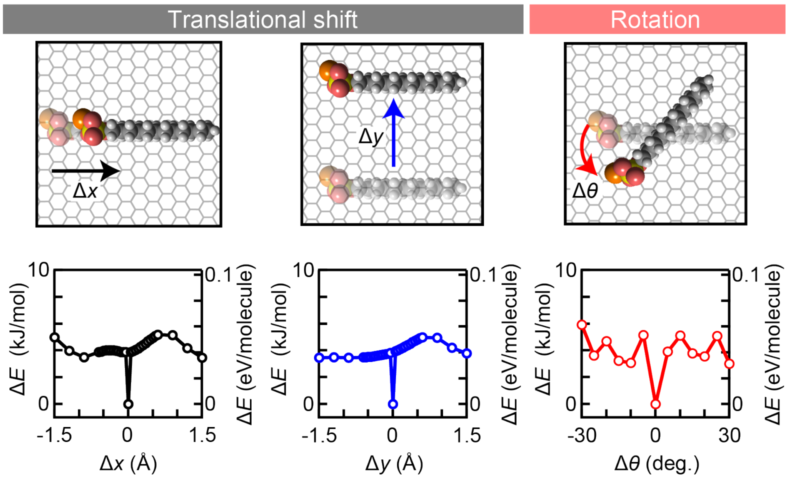


**Supplementary Figure S5.** Schematic of the simulated molecular configurations with the molecules' longitudinal along the armchair direction (upper panels). Calculated destabilization energies ($\Delta E$) as functions of the in-plane translational shifts ($\Delta x$ and $\Delta y$) and the rotation angle ($\Delta\theta$) of the SDS molecule (lower panels).

**Supplementary Table S1**. $\theta_{rr}$ Distribution Detail

| $\theta_{rr}$ **Value (Range)** | **Counted Number** | **Relative Frequency** |
|---|---|---|
| 0° (0° < $\theta_{rr}$ ≤ 10°) | 1 | 0.029 |
| 20° (10° < $\theta_{rr}$ ≤ 30°) | 2 | 0.059 |
| 40° (30° < $\theta_{rr}$ ≤ 50°) | 3 | 0.088 |
| 60° (50° < $\theta_{rr}$ ≤ 70°) | 24 | 0.706 |
| 80° (70° < $\theta_{rr}$ ≤ 90°) | 4 | 0.118 |

Note: The total number of $\theta_{rr}$ data points is 34.

**Supplementary Notes**

**Supplementary Note 1: Direct observation of the formation of SDS ribbons**

Supplementary Figure S2a shows the AFM images of the region indicated by the rectangle in Supplementary Fig. S2b at time $t = 0$, 60, and 120 s, as measured under the intense tip scanning with a tip–sample force of 30 pN (for quasi-real-time morphology change, see Supplementary Movie S1). The initial state ($t = 0$ s) was the condition immediately after that of Fig. 1c in the main text. At $t = 60$ s, the height on the graphene decreased (approximately 0.6 nm, see Supplementary Fig. S2c). This decrease in height indicates that the intense tip scanning removed the initially adsorbed SDS molecules. Afterwards, the SDS ribbons spontaneously grew on the graphene surface at the duration between 60 s and 120 s (the growth of the structure can be clearly seen in Supplementary Movie S1).

**Supplementary Note 2: Inner structure of the SDS ribbons**

Here, we discuss the inner structure of the SDS ribbons. The height of this SDS ribbon is approximately the same along its longitudinal direction (Fig. 1e), and it is on the order of 1.5 nm (Fig. 1f), which is similar to the size of SDS molecule (approximately 2 nm). A similar but periodic (not isolated) one-dimensional aggregation pattern of the SDS molecules on a graphite was observed under high-density conditions close to the critical micelle concentration[1], as predicted by molecular dynamics simulations[2-5]. In these reports, the cross-sections of the SDS aggregations were attributed to a characteristic hemicylinder-like structure in which the hydrophobic end of the SDS molecules faces the graphene surface, whereas the hydrophilic end interacts with water (inset of Fig. 1f).

We fit the cross-sectional profile of the ribbon shown in Fig. 1f by assuming a hemicylinder structure and taking the AFM tip size into account. Supplementary Figure S3 shows a schematic for this analysis. The hemicylinder radius was evaluated as $r \sim 1.3$ nm, with a probe tip radius of $R \sim 20$ nm. This hemicylinder radius is on the order of the length of the SDS molecules. We thus attribute the observed SDS ribbons to a hemicylinder-like nanostructure composed of SDS molecules.

**Supplementary Note 3: The distribution of the ribbon–ribbon angle**

We studied the distribution of the angle between the ribbons (the ribbon–ribbon angle $\theta_{rr}$) from the results of 22 individual AFM experiments. The distribution of the $\theta_{rr}$ value is summarized in Supplementary Table S1.

**Supplementary Note 4: Effect of scan direction and scan rate**

We tested the possible effect of scan direction and scan rate to SDS ribbons already grown on graphene surface. At first, AFM scan direction was 45-degree clockwise rotated with respect to original horizontal direction, while scan rate (4.3 μm/s) and other parameters were kept same. Subsequently, scan rate was further increased to 55.7 μm/s while scan area was manually reduced. The high-speed scanning lasted for 200 s. The direction of molecular assemblies remained unchanged as either scan direction or scan rate changed (Supplementary Fig. S4), indicating SDS ribbons on graphene surface were robust.

**Supplementary Note 5: Computational details**

We optimized a model of an SDS molecule and a graphene consisting of 219 carbon hexagonal rings and terminated by hydrogen atoms. After the optimizations, the SDS molecule was located on the graphene model in either zigzag orientation or armchair orientation; the SDS–graphene was then optimized again, as shown in Figs 3c and Supplementary Fig. S4. The zigzag orientation is more stable than the armchair orientation by 4.0 kJ/mol (42 meV/molecule). We estimated the destabilization energy by a translational shift of the SDS from each most stable geometry in either the zigzag direction or armchair direction or by rotation of the SDS. The relative energies at each shifted orientation were evaluated by single point calculations. The destabilization was evaluated as the relative energy to each optimized structure. Notably, the absolute energy at the zero shift or rotation for the zigzag configuration (Fig. 3c) is smaller by 4.0 kJ/mol (42 meV/molecule) than those for the armchair case (Supplementary Fig. S5). All calculations were performed using the self-consistent charge density-functional tight-binding (SCC-DFTB)[6] with the third-order expansion[7] and the 3ob parameter set[8,9] as implemented in the DFTB+ package, version 1.3.1[10]. The D3 Grimme-type dispersion was included in the all of the calculations[11,12].

In the armchair configuration, an SDS molecule oriented parallel to the armchair direction is unstable even against a very small translational shift (Supplementary Fig. S5), which is in contrast to the zigzag case (Fig. 3c). However, both cases show a similar dependence against the rotational shift.

**Supplementary Note 6: Calculation method for tip–sample force**

Based on the literature[13], average tip–sample force ($f_{ts}$) can be estimated as:

$$f_{ts} = \frac{k}{2Q}\sqrt{A_0^2 - A_s^2}$$

where $A_0$ is the free oscillation amplitude of the cantilever proportional to the drive amplitude ($V_0$, set as 0.1 V in our experiment), and $A_s$ is the amplitude setpoint proportional to the setpoint voltage ($V_s$). $A_0$ ($A_s$) can be calculated as the product of $V_0$ ($V_s$) and the tip-dependent deflection sensitivity (typically ~ 25 nm/V). Tip–sample force was tuned through changing the $V_s$ in the experiment. $Q$ and $k$ are the AFM probe's quality factor and spring constant, respectively. The $Q$ factor was evaluated by performing a frequency sweep in fluid, and the final value was an average of three individual measurements. The $k$ value of the cantilever in use was determined by thermal tune method using its average deflection sensitivity under room temperature (25 °C).

**References**


1. Wanless, E. J. & Ducker, W. A. Organization of sodium dodecyl sulfate at the graphite−solution interface. *J. Phys. Chem.* **100**, 3207–3214 (1996).
2. Domínguez, H. Self-aggregation of the SDS surfactant at a solid−liquid interface. *J. Phys. Chem. B* **111**, 4054–4059 (2007).
3. Sammalkorpi, M., Panagiotopoulos, A. Z. & Haataja, M. Structure and dynamics of surfactant and hydrocarbon aggregates on graphite: a molecular dynamics simulation study. *J. Phys. Chem. B* **112**, 2915–2921 (2008).
4. Domínguez, H. Structural transition of the sodium dodecyl sulfate (SDS) surfactant induced by changes in surfactant concentrations. *J. Phys. Chem. B* **115**, 12422–12428 (2011).
5. Tummala, N. R., Grady, B. P. & Striolo, A. Lateral confinement effects on the structural properties of surfactant aggregates: SDS on graphene. *Phys. Chem. Chem. Phys.* **12**, 13137–13143 (2010).
6. Elstner, M. *et al.* Self-consistent-charge density-functional tight-binding method for simulations of complex materials properties. *Phys. Rev. B* **58**, 7260–7268 (1998).
7. Yang, Y., Yu, H., York, D., Cui, Q. & Elstner, M. Extension of the self-consistent-charge density-functional tight-binding method: third-order expansion of the density functional theory total energy and introduction of a modified effective Coulomb interaction. *J. Phys. Chem. A* **111**, 10861–10873 (2007).
8. Gaus, M., Goez, A. & Elstner, M. Parametrization and benchmark of DFTB3 for organic molecules. *J. Chem. Theory Comput.* **9**, 338–354 (2013).
9. Kubillus, M., Kubař, T., Gaus, M., Řezáč, J. & Elstner, M. Parameterization of the DFTB3 method for Br, Ca, Cl, F, I, K, and Na in organic and biological systems. *J. Chem. Theory Comput.* **11**, 332–342 (2015).
10. Aradi, B., Hourahine, B. & Frauenheim, T. DFTB+, a sparse matrix-based implementation of the DFTB method. *J. Phys. Chem. A* **111**, 5678–5684 (2007).
11. Grimme, S., Antony, J., Ehrlich, S. & Krieg, H. A consistent and accurate ab initio parametrization of density functional dispersion correction (DFT-D) for the 94 elements H-Pu. *J. Chem. Phys.* **132**, 154104 (2010).
12. Grimme, S., Ehrlich, S. & Goerigk, L. Effect of the damping function in dispersion corrected density functional theory. *J. Comput. Chem.* **32**, 1456–1465 (2011).
13. Rosa, L. G. & Liang, J. Atomic force microscope nanolithography: dip-pen,

nanoshaving, nanografting, tapping mode, electrochemical and thermal nanolithography. *J. Phys. Cond. Mat.* **21**, 483001 (2009).
